# NAVIGATING PAYMENT, POLICY, AND HEALTH SYSTEMS: HOW THE ORGANIZATION OF PAID CARE AFFECTS WORKERS AND FAMILIES

**DOI:** 10.1093/geroni/igae098.0390

**Published:** 2024-12-31

**Authors:** Emily Franzosa

**Affiliations:** Chair

## Abstract

The complex structure of paid personal and home care services can cause confusion and frustration for families as they navigate multiple payers and providers to get the care they need. At the same time, the fragmented industry structure, combined with low reimbursement for paid care services, leads to poor quality jobs and high workforce turnover for paid caregivers themselves. While the COVID-19 pandemic laid bare existing fault lines in paid care delivery, it also suggested promising avenues for intervention. This symposium explores organizational factors that may improve stability of the paid care workforce. First, we explore the impact of wages. Miller et al describe the impact of state policies such as hazard pay in stabilizing the workforce shortage during COVID-19, while Jiyeon Kim’s innovative analysis examines whether holding multiple direct care jobs, which is common given the often part-time nature of paid care work, can lift workers out of poverty. Next, Patricia Kim and colleagues use integrated electronic medical record and interview data to explore the organization of paid care at end of life through the experience of patients receiving hospice care during the pandemic. Finally, Walkner et al share a promising pilot in the Veterans Health Administration to integrate paid caregivers into health systems by hiring them directly into health care teams. Together, this research highlights innovative avenues to improve pay, job stability and other key measures that may stabilize the workforce and reduce complexity in the paid care system.

